# Carapace Morphological Characteristics of Chinese Mitten Crab (*Eriocheir sinensis*) from Emerging Origins Revealed via Geometric Morphometrics

**DOI:** 10.3390/ani15091300

**Published:** 2025-04-30

**Authors:** Wang Zhang, Junren Xue, Li Ma, Jian Yang

**Affiliations:** 1 Wuxi Fisheries College, Nanjing Agricultural University, Wuxi 214100, China; 2023113020@stu.njau.edu.cn (W.Z.); xuejunren@ffrc.cn (J.X.); 2 Laboratory of Fishery Microchemistry, Freshwater Fisheries Research Center, Chinese Academy of Fishery Sciences, Wuxi 214081, China; 3 Xinjiang Uygur Autonomous Region Fisheries Research Institute, Urumqi 830099, China; xe_1218@163.com

**Keywords:** Chinese mitten crab, emerging origins, geometric morphometrics, landmark

## Abstract

This study used geometric morphometrics to investigate the carapace features of Chinese mitten crabs (*Eriocheir sinensis*) from non-traditional/emerging origins (Zhenlai County and Bosten Lake) and identify differences in features relative to crabs from traditional origins (Yangcheng Lake). Thirty-five landmark data points on the crab carapace were extracted based on the geometric morphometrics landmark point approach and standardized to obtain the mean shape of the crabs from the three origins. The carapace morphologies of crabs from the three different origins varied. Compared to the average morphology, the first lateral tooth of the carapace of the Zhenlai crab was shorter, and the M pattern was enlarged in males, whereas the first and second lateral teeth of the crabs from the other two origins were longer, and the M pattern was reduced. In female crabs, distinct morphological variations were observed across regions. In Zhenlai crabs, the lateral teeth were shorter, and the M pattern was enlarged. In Bosten crabs, the frontal tooth was shorter, the first and second lateral teeth were longer, and the posterior carapace margin was narrowed. In Yangcheng Lake crabs, the frontal and third lateral teeth were elongated, and the M pattern was narrowed. The discrimination accuracy of individual female and male crabs from different origins reached 100%. The results confirm that the carapace geometric morphometric method represents a convenient and non-lethal method of tracing *E. sinensis* origins in fine-scale production areas. Thus, this study provides guidance on developing a characteristic industrial chain for aquatic products of emerging origins, which may help to promote the sustainable development of the aquaculture industry in associated areas.

## Simple Summary

The present study was performed to comparatively analyze morphological variations in the carapace shape of *Eriocheir sinensis* populations from non-traditional/emerging origins (Bosten Lake and Zhenlai County) and traditional origins (Yangcheng Lake) using a geometric morphometric method. A combination of thin-plate spline analysis and generalized Pratt’s analysis was applied to the morphometric study of these valuable crabs. Different geographic populations showed significant spatial heterogeneity in carapace morphology. Geometric morphometrics based on carapace landmarks have a discrimination accuracy of up to 100% for male and female crabs from different production areas and can thus provide a more convenient and non-lethal method of tracing the origins of Chinese mitten crabs in fine-scale production areas. It can also be used to form a characteristic industrial chain for aquatic products of new origins and alleviate poverty in remote rural areas, which in turn will promote the sustainable development of the aquaculture industry.

## 1. Introduction

Chinese mitten crab *(Eriocheir sinensis*, commonly known as hairy crab or river crab) is distributed in rivers, lakes, and reservoirs in eastern China and Southeast Asia and represents a pivotal species in China’s aquaculture industry [1]. It is not only a traditional and valuable food but also embodies a wealth of gastronomic and cultural significance and historical inheritance. These crabs have high nutritional value and are rich in proteins, trace elements, and many vitamins [2,3]. Crab products in traditional production areas such as Yangcheng Lake in Suzhou are known for their excellent quality and significant output value. Moreover, the hairy crabs of Yangcheng Lake have long adapted to the weakly alkaline water and sandy substrate conditions of the lake area and evolved an iconic phenotype that includes a ‘green back, white belly, golden claw, and yellow hair’ [4,5]. These features not only represent a biological ‘identity card’ of authenticity for consumers but also elevate the market competitiveness of crabs from Yangcheng Lake, which are several times more expensive than crabs originating from other regions. The morphological characteristics of aquatic products are deeply bound to their environment of origin, which directly influences consumers’ choice and their economic value. With the promotion of national rural revitalization and local economic policies, the range of *E. sinensis* cultivation has expanded [6]. In recent years, crab industries of emerging origins (i.e., non-traditional and newly developed production areas that have rapidly expanded beyond traditional farming regions through technological innovation, ecological adaptation, or regional resource integration), represented by Bosten Lake in the Xinjiang Uygur Autonomous Region (hereinafter referred to as Xinjiang Province) and Zhenlai in Jilin Province, have gradually increased in popularity. Taking advantage of the unique local natural resources and environment and scientific culture management methods, *E. sinensis* aquatic products of regional reputation have been successfully cultured and gradually gained a foothold in the market. The rise of products from new origins has not only injected new vitality into local economic development but also provided consumers with more valuable choices. Moreover, innovations have been realized in *E. sinensis* aquaculture, which plays an important role in rural revitalization. Because the growth and development of aquatic animals are strongly influenced by environmental factors, water quality, temperature, bait resources, and culture techniques from different origins may lead to significant differences in the animal’s morphological characteristics [7,8]. Furthermore, the morphological profile or identification benchmark of *E. sinensis* from emerging origins can be established using their different morphological characteristics to improve the recognition and economic value of hairy crabs from emerging origins. Therefore, in the face of increasingly diversified culture environments, differences in morphological quality among *E. sinensis* from traditional and emerging origins should be established using Yangcheng Lake hairy crabs of traditional origin as a reference. The results will not only help reveal the influence of environmental factors on the growth and development of *E. sinensis* but also highlight the habitat characteristics of the two emerging origins.

Several methods are available for biomorphological studies, including geometric morphometrics, which is a mathematical and statistical methodology used to accurately analyze morphological variations in organisms. Significantly adaptive marker points have been selected and positioned to obtain their 2D or 3D coordinates [9,10], and geometric transformations (translations, rotations, and scaling) are then applied to align marker points across individuals, thereby eliminating non-morphological variations. This method can then be used to explore patterns of morphological variation using statistical methods (e.g., PCA) [11,12,13]. In recent years, geometric morphometry has been increasingly used in the study of aquatic biomorphology, especially in the study of vertebrates, such as gobiid fishes [14], and invertebrates, such as crabs [15]. In addition, Dwivedi et al. [16] used geometric morphometrics to assess the phenotypic relationships among 39 Gangetic catfish species by analyzing photographs of the species. Morphospatial phenograms indicated that all species within the same genus were closely clustered, revealing shared phenotypic traits and evolutionary closeness of species within the genus. Traverso et al. [17] applied two geometric morphometric methods, landmark- and contour-based, to the scales of five different fish species and found that both methods were effective for the cluster analysis of all species. Moreover, their study demonstrated that the rapid and economical identification of fish species on a single scale is feasible and important in a variety of contexts. Graham et al. [18] investigated whether the claws of female Allegheny crayfish (*Faxonius obscurus*) change in size or shape according to the reproductive state of the animal by using 5 landmark points and 16 half-landmark points on the claws to describe their shape and analyze their geometric morphometrics, respectively. The results showed that during the reproductive season, crayfish had larger claws but showed little difference in claw shape. Farré et al. [19] performed the first study to demonstrate the importance of the carapace shape on the invasive ability of marine decapod crustaceans using a model based on geometric morphometrics. The results suggested that invaders with extreme carapace features (e.g., *Callinectes sapidus*) are located at the periphery of the community’s morphological space and are usually ecologically dominant, which contributes to their success. In contrast, intermediate forms within the morphological space (e.g., *Dyspanopeus sayi*) imply ecological overlap with native species, which reduces their community relevance. Morphometric approaches also represent additional tools for assessing the potential capacity, translating morphological differences into quantitative indicators of ecological function, and revealing the mechanisms underlying the competitive advantage of invasive crab species. To study shape differences between *Chamelea gallina* and *Chamelea striatula* in Portuguese coastal waters, Rufino et al. [20] used contour (elliptical Fourier analysis) and landmark point-based methods. The shape variables provided by the geometric morphometric method resulted in a 0–6% misclassification rate between species. However, the contour analysis showed that the differences between species were mainly in the umbo and lunular areas of the shell, for which they created an index and successfully used it to distinguish between species (100% correct classification). In conclusion, the geometric morphology approach represents a scientific and systematic analysis tool that provides precise and quantitative data on the morphological features of aquatic animals, thereby greatly facilitating a detailed analysis of inter- and intraspecific morphological differences.

Therefore, the present study was performed to systematically analyze morphological variations in the carapace structure of *E. sinensis* from different geographic populations using geometric morphometric methods. By comparing morphological differences between crabs of emerging origins, represented by Bosten Lake in Xinjiang Province and Zhenlai County in Jilin Province, and crabs of traditional origin, represented by Jiangsu Yangcheng Lake, we aimed to construct a crab morphological method based on landmark points to expand the characteristics of crustacean morphometrics and develop a benchmark for discriminating the morphological quality of aquatic species of economic importance. In this study, a combination of thin-plate spline analysis and generalized procrustes analysis was applied to the morphometric study of freshwater crabs. This method not only compensated for the shortcomings of traditional linear measurements in terms of overall morphometric resolution [21] but also provided a geometric topology-based quantitative discriminative system for the assessment of aquatic germplasm resources.

## 2. Materials and Methods

### 2.1. Experimental Crabs

**Figure 1:**
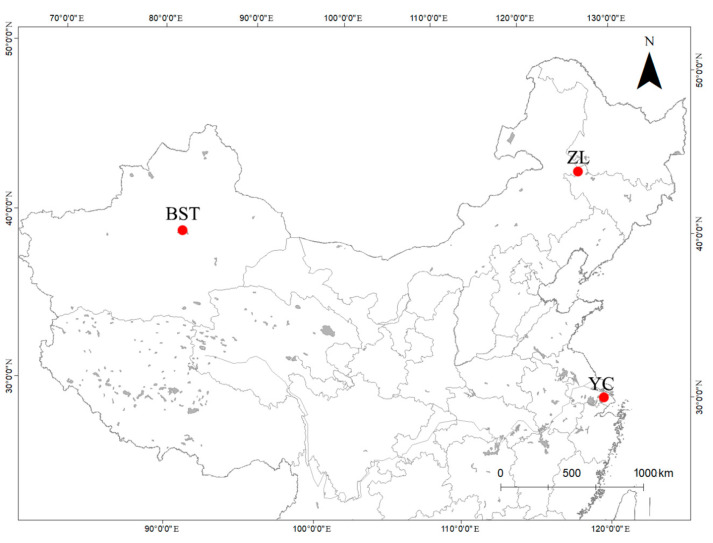
Sketch map of sampling locations for *Eriocheir sinensis* in China. ZL, BST, and YC represent sample locations from Zhenlai County (Jilin Province), Bosten Lake (Xinjiang Province), and Yangcheng Lake (Jiangsu Province), respectively.

Samples of *E. sinensis* were collected in September 2024 from the aquaculture waters of Zhenlai County (Jilin Province), in October 2024 from the aquaculture waters of Bosten Lake (Xinjiang Province), and in November 2024 from the waters of Kunshan Yangcheng Lake (Jiangsu Province) (Figure 1). The samples were collected and brought back to the laboratory for morphometric measurements (Table 1), after which they were stored in a −20 °C refrigerator. A total of 20 *E. sinensis* crabs (male and female, 10 each) of similar size were selected for each sample group. All crabs met the requirements of native crabs (i.e., kept in the same water from the time they were raised from juvenile crabs to adult crabs) according to national standard GB/T19957-2005 [22] for *E. sinensis* native to Yangcheng Lake.

### 2.2. Methods

#### Establishment and Extraction of Landmarks

**Figure 2:**
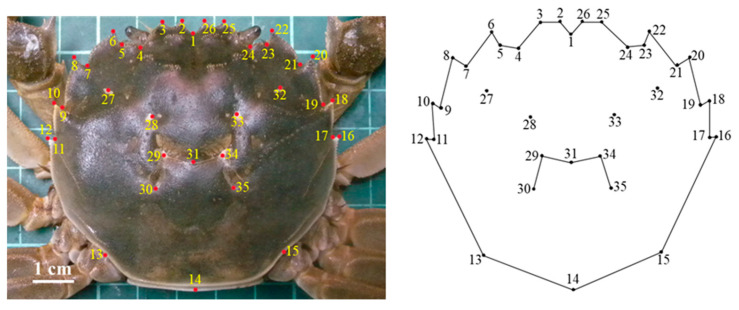
Locations of carapace 35 landmarks on *Eriocheir sinensis* specimens.

Zheng et al. [23] compared the carapace and breastplate characteristics of *E. sinensis* using the geometric morphometric analysis method. The breastplate characteristics were inadequate to differentiate *E. sinensis* females and males from different origins, which had different shapes, and averaged 83.75% and 98.75% in accuracy, respectively. However, the carapace characteristics could well differentiate females and males of different origins, which had similar shapes and achieved 100% accuracy. Therefore, only the carapace was used for geometric morphometric characterization and comparisons based on the landmark points in this study. Landmarks are points at locations with distinctive features that are easily recognizable, and they play a crucial role in morphological studies. Landmarks can be divided into three main categories: type I, which is characterized by intersections between different tissue structures; type II, which is characterized by depressed or raised points in the structure; and type III, which is characterized by structural poles [24]. To standardize the landmark point data, a digital camera (COOLPIX P6000, Nikon, Tokyo, Japan) was used to capture pictures of the crab carapace at a fixed shooting height and focal length. A fixed mounting system was employed to maintain consistent camera-to-subject distance and pitch angle throughout the imaging process. Reference lines were clearly marked on the background to prevent positional deviations, and a standardized green grid panel was uniformly implemented as the background substrate. These images were then imported into tpsDig2 2.32 software [25,26] for processing to ensure uniform scaling across the dataset, with the preprocessing phase completed prior to landmark annotation. Thirty-five landmark points on the carapace were representatively and precisely selected according to the principle of landmark point classification (Figure 2 and Table 2), and their X and Y coordinate values were accurately recorded to create consistent data files of landmark points.

### 2.3. Geometric Morphological Analysis

A least-squares regression analysis was implemented in tpsSmall 1.36 software to assess the reliability of the landmark points. Next, a series of geometric processing steps, including Platonic superposition, were performed using tpsRelw 1.75 [27] and PAST software (5.0) [28] to calculate the center-of-mass distances between landmark points. The results were then used to derive the mean shape and matrix of relative distortion indices and generate a comprehensive data report. In addition, thin-plate spline analysis was performed with the aid of PAST software to generate a hotspot grid map of the *E. sinensis* carapace. This map was used to identify and compare the characteristics of the morphological variability of the carapace in the crabs from different origins. Namely, biologically meaningful landmarks (e.g., distinct protuberances or depressions along the carapace margins) were carefully selected to ensure anatomical consistency. Over 35 landmark points were digitized to comprehensively cover key regions of the carapace, including the lateral teeth, frontal margins, and mid-gill area, thereby minimizing errors from localized deformations. Image normalization and landmark coordinate extraction were performed using geometric morphometric software from the TPS series. The TPS algorithm models shape deformation by minimizing the bending energy of a hypothetical thin plate, mathematically represented as the integral of second-order derivatives. This analysis was conducted using tpsRegr 1.50 [29] software. This process ensured smooth and biologically plausible transformations. To facilitate more intuitive visualization of morphological changes, the same data were subsequently analyzed via PAST software. The congruence of results between both methods validated our findings, and these outcomes were therefore retained in the final analysis.

### 2.4. Discriminant Analysis

SPSS 27.0 software (IBM Corp., Armonk, NY, USA) was used to perform discriminant analysis on the relative warp scores of each sample by applying the Bayes method [30].

## 3. Results

### 3.1. Visual Difference Analysis of Carapace Morphology

**Figure 3:**
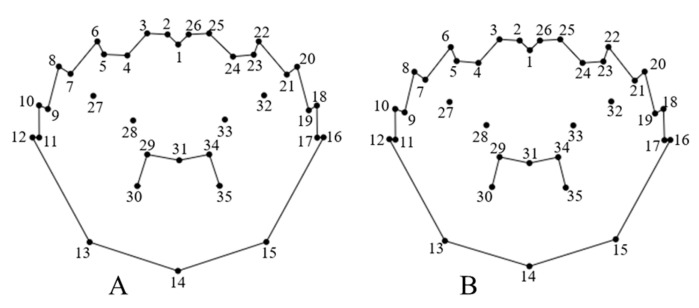
Mean shape of carapace of *Eriocheir sinensis* with landmark numbers. (**A**) Male; (**B**) female.

The regression coefficients of distance in tangent space (*y*-axis) and the Procruste distance (*x*-axis) for the carapace landmarks of male and female Chinese mitten crabs from different origins were 0.999928 and 0.999940, respectively. These values are close to 1, suggesting that the landmarks selected in this study are valid and could be applied for subsequent geometric morphology analysis and related statistical analyses. The carapace landmark points of *E. sinensis* from the three origins were averaged using PAST software to obtain the mean shape of the carapace morphology (Figure 3). In addition, in the landmark point analysis of the carapace landmark point data of *E sinensis* from the three origins, the contribution rates of different landmark points differed, which indicated the variability of morphological points for crabs from the three origins. The higher the contribution rate, the higher the variability of the point. Among them, the 11th, 12th, 16th, and 17th landmark points of male and female crabs had the highest contribution rates. In the male and female crabs, these landmark points explained 49.37% and 48.12% of the morphological variation, respectively, while the other 31 landmark points explained 50.63% and 51.88% of the variation, respectively.

**Figure 4:**
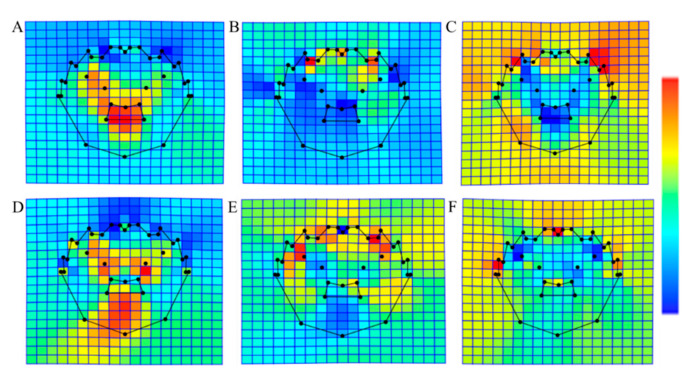
The deformation of carapace lattice hotspots in *Eriocheir sinensis*. (**A**) Zhenlai male crabs; (**B**) Bosten male crabs; (**C**) Kunshan Yangcheng Lake male crabs; (**D**) Zhenlai female crabs; (**E**) Bosten female crabs; (**F**) Kunshan Yangcheng Lake female crabs. Red indicates that the area is enlarged relative to the average profile, blue indicates that the area is reduced relative to the average profile, and green indicates that it is close to the average profile.

The mean values of the 35 landmark points on the carapaces of male and female crabs from the three origins were analyzed using the thin-plate spline method to obtain images of any relative distortions with respect to their mean values (Figure 4). Figure 4 shows that all three species of crabs showed increases and decreases relative to their mean values at different landmark points. For male Zhenlai crabs, the left middle gill area (27th and 28th landmark points) and the middle of the M pattern (29th, 30th, 31st, 34th, and 35th landmark points) expanded outwards, while the frontal margins, the concavity between these margins and first lateral teeth (3rd and 4th landmark points), and first lateral teeth on the right side (22nd and 23rd landmark points) narrowed inwards. In male Bosten Lake crabs, the concave point between the frontal margin and first lateral tooth and the medial apex of the first lateral tooth (4th, 24th, 5th, and 23rd landmark points) expanded outwards, and the left median gill area (27th and 28th landmark points), the middle of the M pattern (29th, 30th, 31st, 34th, and 35th landmark points), and right second lateral tooth (20th and 21st landmark points) narrowed inwards. In male Yangcheng Lake crabs, landmark points on the frontal margin and lateral teeth on the outside expanded outwards, while the landmark points on the mid-cheek area and the M pattern on the inside narrowed inwards. In female Zhenlai crabs, the mid-gill area (27th, 28th, 33rd, and 32nd landmark points) and lower apex of the M pattern (30th and 35th landmark points) expanded outwards, while the frontal margins (2nd, 3rd, 26th, and 25th landmark points), right first lateral tooth (22nd and 23rd landmark points), and third lateral tooth (9th, 10th, 18th, and 19th landmark points) narrowed inwards. In female Boston Lake crabs, the concavity between the frontal margin and first lateral tooth (27th and 32nd landmark points) and concavity between the two foramina and second lateral tooth in the mid-cheek area (4th and 24th landmark points) expanded outwards, and the frontal margin and its center (1st, 2nd, and 26th landmark points) and lower apex of the M pattern (30th and 35th landmark points) narrowed inwards. In female Yangcheng Lake crabs, the frontal margin and its center (1st, 2nd, and 26th landmark points) and third lateral tooth (9th, 10th, 18th, and 19th landmark points) all extended outwards, and the concave points between the two holes in the mid-cheek area and second lateral tooth and the inner apex of the second lateral tooth (27th, 32nd, 7th, and 21st landmark points) narrowed inwards.

### 3.2. Discriminant Analysis of Morphological Differences in Carapace

**Figure 5:**
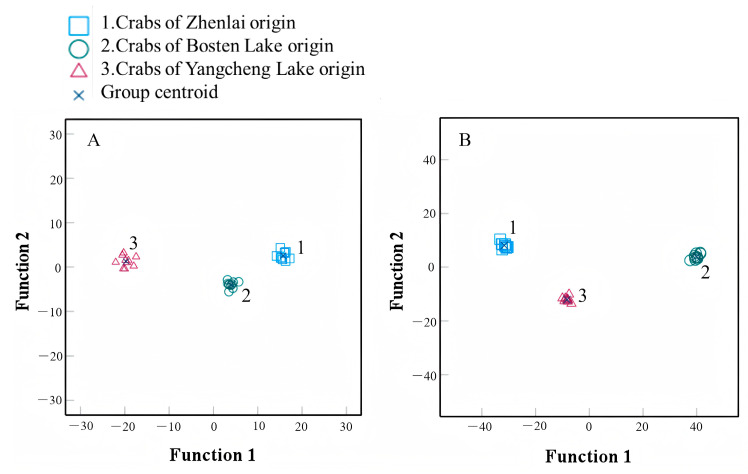
Discriminant analysis of carapace geometry of *Eriocheir sinensis* from different origins illustrated via scatter diagram. (**A**) Male; (**B**) female.

The carapaces of both male and female crabs were discriminated among the three origins with an accuracy of 100% (Table 3). The scatter plot of the discriminant analysis (Figure 5) showed the crab carapace morphology more intuitively, and the high discrimination accuracy of the proposed landmark method revealed the effectiveness of the method.

## 4. Discussion

### 4.1. Inter-Origin Differences in Carapace Morphology

In this study, we used the geometric morphometrics of landmark points to analyze the morphology of *E. sinensis* from emerging origins. The results show that different geographic populations presented significant differences in carapace morphological characteristics. Specifically, Zhenlai County in Jilin Province showed an expanding trend in the middle gill area and M pattern area inside the carapace of both sexes, while the frontal margin and the first lateral tooth showed contraction; the Bosten Lake population in Xinjiang Province showed the opposite morphology, with a significant contraction in the M pattern area but an expanding trend in the frontal margin and the first lateral tooth; and the Yangcheng Lake population showed a unique morphology, with a significant contraction in the gill area and contraction in the M pattern area in males but a general expansion in the lateral tooth area in both sexes. The morphological classification model established by discriminant analysis showed a high degree of geographic differentiation (100% discriminant accuracy), confirming the significant correlation between the morphological characteristics of the carapace of *E. sinensis* and its geographic origin. Although the causal mechanisms of morphological differences have not been clarified, this study speculates that multidimensional influences may be involved. From a genetic perspective, parental germplasm traits and seedling selection may result in genetic differentiation [31]. From an environmental perspective, the unique hydrochemical characteristics (e.g., pH and ionic composition), hydrological conditions (water temperature and flow rate), and bait composition of various production areas may induce phenotypic plasticity. Specifically, the expansion of the frontal margin and contraction of the mid-gill area of Yangcheng Lake crabs may represent long-term adaptations to increased competition for feeding in eutrophic waters. In contrast, the growth of Zhenlai crabs was likely slowed by the low temperatures of the cold freshwater area in Jilin Province, while differences in the rate of localized calcification of the carapace of Bosten Lake crabs were likely related to the effects of high-salinity osmotic stress caused by the high-saline lake environment [32,33,34,35]. From a culture practice perspective, differentiated culture modes (feeding strategies and culture densities) and management measures may influence morphological development through growth regulation [5]. Follow-up studies are needed to integrate genomics, environmental genomics, and experimental data from aquaculture to analyze these mechanisms in multiple dimensions.

### 4.2. Potential Application of Morphological Characteristics for Geographical Origin Protection

In this study, a landmark point analysis of the carapace of *E. sinensis* from three origins showed that the landmark points with the highest contributions were the 11th, 12th, 16th, and 17th landmark points in male and female crabs. Therefore, these landmarks play a key role in characterizing geomorphological differences. Notably, the discriminant model constructed based on landmark points demonstrated excellent origin traceability based on a discriminant accuracy of 100%, which was substantially higher than that of traditional morphometric methods, e.g., ca. 80% accuracy obtained by morphological truss network analysis [36]. This finding is corroborated by recent research in the field of morphometry. Liu et al. [37] established a dataset containing three traditional morphometric (TMM) attributes and a dataset containing 18 geometric landmark points in *Crassostrea gigas* and *C. ariakensis*, which were then subjected to a variety of analyses to determine whether differences occurred between the two oyster species. The results revealed that the dataset utilizing geometric landmark points demonstrated a significant advantage and presented a classification accuracy of 100%, which far exceeded the ambiguous classification results due to morphological overlap in traditional morphometric methods. Thus, the morphological characteristics of aquatic products are substantially correlated with their geographic origin. Therefore, we used the morphological characteristics of crabs from each place of origin to establish a morphological discrimination benchmark for aquatic products. Through these identification benchmarks, the phenomenon of origin impersonation can be effectively curbed, and the brand reputation of emerging origins can be maintained, thus increasing the recognition and economic value of aquatic products from major emerging origins. Finally, a double breakthrough was achieved in this study at both the theoretical and applied levels because the differences in morphological characteristics between crab populations from arid (Xinjiang Province) and humid zones (Jilin Province) were quantified for the first time, which revealed the effects of different geographic environments on morphological differentiation. In addition, the morphological characteristics of crabs from different emerging origins provided reliable identification criteria for the certification of aquatic products as geographical indications. Follow-up studies may combine genome association analysis to locate the key regulatory loci of morphological characteristics and analyze the mechanism underlying the effects of temperature–salinity gradients on carapace development through controlled experiments. Such work will provide theoretical support for the improvement in conservation regarding endemical germplasm resources and the development of a special automate carapace landmark-based detection system for origin identification for *E. sinensis*.

## 5. Conclusions

In this study, 35 landmark points and geometric morphometric characteristics of male and female Chinese mitten crab samples from three typical production areas, namely Zhenlai in Jilin Province, Bosten Lake in Xinjiang Province, and Yangcheng Lake in Jiangsu Province, were used to establish a two-dimensional coordinate system. The results revealed that different geographic populations showed significant spatial heterogeneity in carapace morphology and that the geometric morphometrics based on the carapace landmarks had a discrimination accuracy of up to 100% for male and female crabs from the different production areas. The carapace geometric morphometric method can provide a more convenient and non-lethal technical method of tracing the origins of Chinese mitten crabs in fine-scale production areas. Moreover, this method can also be used to promote special endemical brands for aquatic (especially *E. sinensis*) products of emerging origins and alleviate poverty in remote rural areas, which in turn will promote the sustainable development of the aquaculture industry. Based on these research findings, we will further expand the sampling scope of these two emerging localities in subsequent experiments to obtain more accurate carapace characteristics while systematically exploring the formation mechanisms underlying these regional-specific features.

## Figures and Tables

**Table 1:** Morphometric information of Chinese mitten crab (*Eriocheir sinensis*) populations from three different origins.

Geographical Origin	Sampling Time	Gender	Sample Size	Body Weight/g	Carapace Length/mm	Carapace Width/mm	Body Height/mm
Zhenlai (Jilin Province)	2024.09	Male	10	135.65 ± 6.64	60.60 ± 0.70	67.82 ± 1.07	32.36 ± 1.17
		Female	10	112.68 ± 7.92	59.67 ± 1.07	65.04 ± 1.84	33.26 ± 1.02
Bosten Lake (Xinjiang Province)	2024.10	Male	10	116.96 ± 9.72	57.86 ± 0.72	66.75 ± 1.33	31.55 ± 0.94
		Female	10	88.17 ± 3.56	55.12 ± 0.54	61.69 ± 1.01	31.15 ± 0.85
Yangcheng Lake (Jiangsu Province)	2024.12	Male	10	145.81 ± 6.86	61.41 ± 0.77	68.92 ± 1.24	33.07 ± 0.64
		Female	10	103.90 ± 6.91	57.43 ± 1.50	63.64 ± 1.08	32.51 ± 0.99

**Table 2:** Landmark point types and definitions in the present study.

Type of Landmark Landmark Number		Definition
Type II	2, 3, 25, 26	Frontal margin
	4, 24	Concave spot between frontal margin and first lateral tooth
	5, 23	Base of first lateral tooth
	6, 22	Apex of first lateral tooth
	7, 21	Base of second lateral tooth
	8, 20	Apex of second lateral tooth
	9, 19	Base of third lateral tooth
	10, 18	Apex of third lateral tooth
	11, 17	Base of fourth lateral tooth
	12, 16	Apex of fourth lateral tooth
	13, 15	Rear margin convex point
	27, 32	Concave point in middle gill area between two holes and second lateral tooth
	28, 33	Two holes in middle gills
	29, 34	Vertex on M pattern
	30, 35	Bottom vertex on M pattern
	31	Center of M pattern
Type III	1	Frontal edge center
	14	Medial apex of rear margin

**Table 3:** Discriminant analysis of Chinese mitten crabs (*Eriocheir sinensis*) from three origins based on carapace morphology.

Geographical Origin	Discriminant Accuracy/%		
	Zhenlai of Jilin Province	Bosten Lake in Xinjiang Province	Yangcheng Lake in Jiangsu Province
Zhenlai (Jilin Province)	10/100%	0	0
Bosten Lake (Xinjiang Province)	0	10/100%	0
Yangcheng Lake (Jiangsu Province)	0	0	10/100%

## Data Availability

The data supporting the findings of this study are available from the corresponding author upon reasonable request.
